# Cost‐effectiveness analysis of human papillomavirus (HPV) genotyping strategies for management of HPV‐positive women in cervical cancer screening

**DOI:** 10.1002/ijc.70344

**Published:** 2026-01-28

**Authors:** Kelsi R. Kroon, Johannes A. Bogaards, Johannes Berkhof

**Affiliations:** ^1^ Department of Epidemiology and Data Science Amsterdam UMC, location Vrije Universiteit Amsterdam Amsterdam The Netherlands; ^2^ Methodology Amsterdam Public Health Research Institute Amsterdam The Netherlands; ^3^ Imaging and Biomarkers Cancer Center Amsterdam Amsterdam The Netherlands

**Keywords:** cervical cancer screening, cost‐effectiveness, HPV genotyping, triage of HPV‐positives

## Abstract

In 2017, the Netherlands introduced primary human papillomavirus (HPV)‐based screening with cytology triage, which increased colposcopy referrals and low‐grade lesions detected. In 2022, HPV16/18 genotyping was added for women with borderline/low‐grade cytology. Triage with HPV genotyping may better balance screening benefits, harms, and costs. Therefore, we evaluated the cost‐effectiveness of 19 triage strategies based on net monetary benefit (NMB) at cost‐effectiveness thresholds of €20,000 and €50,000/quality‐adjusted life‐year (QALY), with the highest NMB indicating the most cost‐effective strategy. Triage tests included 16/18 genotyping, 7‐type (16/18/31/33/45/52/58) genotyping, and cytology (high‐grade squamous intraepithelial lesions [HSIL]: moderate/severe, atypical squamous cells of undetermined significance [ASC‐US]/low‐grade squamous intraepithelial lesions [LSIL]: borderline/low‐grade, negative for intraepithelial lesion or malignancy [NILM]: normal). Effects on cancer incidence and mortality were obtained by combining POBASCAM trial data with nationwide screening and cancer registries. Time since the onset of high‐grade lesion (cervical intraepithelial neoplasia [grade 2/3] [CIN2/3]) at baseline of the Population‐based Screening Study Amsterdam trial cannot be estimated from the data and was varied between 0 and 10 years. At a €20,000/QALY threshold, immediate referral for HSIL and 16/18‐positive ASC‐US/LSIL, and repeat cytology for 16/18‐negative ASC‐US/LSIL and 7‐types positive NILM had the highest NMB (€214.1 to €309.3/woman gained compared to referring all HPV‐positive women). At a €50,000/QALY threshold, immediate referral for all 16/18‐positives and/or HSIL, and repeat cytology for 16/18‐negative ASC‐US/LSIL and 7‐types positive NILM had the highest NMB if time since onset of CIN2/3 was greater than 2 years. Cytology combined with HPV16/18 and extended genotyping is cost‐effective for triage of HPV‐positives. Immediate colposcopy referral of all HPV16/18‐positives is cost‐effective at a €50,000/QALY threshold.

## Graphical abstract

What's new?

Persistent infection with high‐risk human papillomavirus (HPV) causes most invasive cervical cancer cases, leading many countries to transition from cytology to primary HPV‐based screening. Despite the benefits, HPV‐based screening may also lead to unnecessary procedures, psychological burden, and strain on healthcare systems. Here, the authors evaluated the trial‐based cost‐effectiveness of 19 HPV genotyping strategies for the management of HPV‐positive women. The results show that triage by cytology combined with HPV16/18 and HPV16/18/31/33/45/52/58 genotyping is cost‐effective compared to cytology triage alone. Triage by HPV16/18 genotyping without adjunct cytology is cost‐effective when the cost‐effectiveness threshold is increased to €50,000 per quality‐adjusted life‐year.

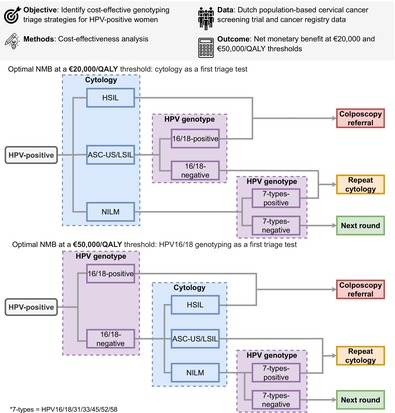

**Abbreviations**
ASCCP: American Society for Colposcopy and Cervical Pathology
ASC‐US: atypical squamous cells of undetermined significance
ASC‐H: atypical squamous cells – cannot rule out high‐grade squamous intraepithelial lesion
CHEERS: consolidated health economic evaluation reporting standards
CI: confidence interval
CIN (2/3+): cervical intraepithelial neoplasia (grade 2/3 or worse)
FIGO: Fédération Internationale de Gynécologie et d'Obstétrique
(hr) HPV: (high‐risk) human papillomavirus
HSIL: high‐grade squamous intraepithelial lesions
LSIL: low‐grade squamous intraepithelial lesions
NCR: Netherlands cancer registry
NILM: negative for intraepithelial lesion or malignancy
NMB: net monetary benefit
NPV: negative predictive value
PALGA: nationwide network and registry of histo‐ and cytopathology in the Netherlands
POBASCAM: Population‐based Screening Study Amsterdam
PPV: Positive predictive value
QALY: quality‐adjusted life‐year

## 1 INTRODUCTION

Persistent infection with high‐risk (hr) human papillomavirus (HPV) causes nearly all invasive cervical cancer cases,^1^ leading many countries to transition from cytology to primary HPV‐based screening.^2^ While HPV‐based screening detects precancerous lesions earlier, it increases the number of colposcopy referrals and low‐grade lesions detected,^3^ resulting in unnecessary procedures, psychological burden, and strain on healthcare systems. To limit the number of unnecessary procedures, HPV‐positive women are commonly triaged by cytology, sometimes in combination with HPV genotyping. HPV genotypes are strongly associated with the risk of precancer (cervical intraepithelial neoplasia grade 3 [CIN3]) and cancer^4^ and many HPV tests provide genotype information either on the individual level or subgroup level.^5^, ^6^, ^7^ The use of genotyping is also particularly relevant for vaccinated cohorts, as HPV‐vaccinated cohorts have a different genotype distribution and a substantially lower cervical cancer risk,^8^ and for HPV self‐sampling cohorts^9^, ^10^ as cytology cannot be performed on self‐collected samples, so HPV‐positive women must get an additional clinician‐collected smear.

Various studies have already compared the performance of HPV genotyping strategies for colposcopy referral. Most of these studies focus on referral rates and positive/negative predictive values (PPV/NPV) for detection of precancer and cancer (CIN3+).^11^, ^12^, ^13^ CIN3+ risk‐based control can lead to high false referral rates, for example, American Society for Colposcopy and Cervical Pathology (ASCCP) guidelines^14^ recommend colposcopy referral at 4% CIN3 risk, implying a false positive rate as high as 96%. Overall, there is a lack of consensus on the use and choice of the risk thresholds. To support policy making, some studies take a health economic approach^15^, ^16^ which determines the most efficient and effective use of limited health resources by calculating the health effects and costs of an intervention. A limitation of these studies is that they rely on the specification of a natural disease progression model over the lifespan of an individual, which is challenging on the level of the HPV genotype.

In this study, we performed a data‐driven, trial‐based cost‐effectiveness analysis of different genotyping strategies for referral of HPV‐positive women to colposcopy. By combining data from the Population‐based Screening Study Amsterdam^17^ (POBASCAM) with nationwide registries, we predicted POBASCAM trial outcomes over two screening rounds for the reference strategy in which all HPV‐positive women were immediately referred for colposcopy and 18 less intensive triage strategies. The POBASCAM study is ideally suited for evaluation of triage strategies because only women with moderate/severe cytological abnormalities (high‐grade squamous intraepithelial lesions [HSIL]) were referred for colposcopy at baseline and other HPV‐positive women were followed by cytology and HPV testing. We assessed the cost‐effectiveness of less intensive strategies by calculating the net monetary benefit (NMB) of each strategy compared to the reference strategy.

## 2 METHODS

**FIGURE 1:**
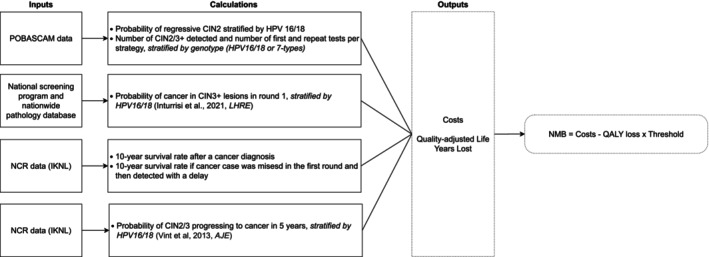
Flow chart of four sources of data used for the analysis^10^, ^18^. CIN (2/3), cervical intraepithelial neoplasia (grade 2/3); HPV, human papillomavirus; IKNL, integraal kankercentrum Nederland; NCR, Netherlands Cancer Registry; NMB, net monetary benefit; POBASCAM, Population‐based Screening Study Amsterdam; QALY, quality‐adjusted life‐year.

Four sources of data were used to assess the cost‐effectiveness of triage strategies in the Dutch cervical cancer screening setting. A summary of the data used is given in Figure 1.

### 2.1 Screening cohort data

We assessed the cost‐effectiveness of triage strategies by comparing the health effects and costs of detecting high‐grade lesions (CIN2/3) and cancer in the baseline round of the POBASCAM study^17^ instead of in the next round after 5 years. The POBASCAM study was a randomized controlled HPV screening trial (registered under ID: NTR218) in the Netherlands with enrolment between 1999 and 2002. The study has been described in detail previously.^17^ This paper specifically focuses on a subset of 1102 HPV‐positive women from the intervention arm who were tested for HPV types 16/18/31/33/35/39/45/51/52/56/58/59/66/68 and had valid cytology results at baseline (start of the first round). For women with abnormal cytology and CIN3+ who violated the protocol (CIN3+ detected before or without repeat test), we imputed the repeat cytology result using the distribution of available repeat test results in women with abnormal cytology and CIN3+ detected in the first round. Since HPV‐positive women with normal cytology in the control arm were not followed in the first round, the probability of CIN2 lesion regression was also estimated with POBASCAM data by comparing the intervention to the control arm over two screening rounds.

### 2.2 Screening registry data

The POBASCAM study had a limited number of cancer cases, so data from the Dutch national HPV‐based screening program (ScreenIT RIVM, Bilthoven, NL) and the nationwide registry of histo‐ and cytopathology in the Netherlands (PALGA, Houten, NL) were linked to calculate the percentage of cancer cases in detected CIN3+ cases at the baseline round,^10^ stratified by HPV16/18. The program data was collected from women who were invited to HPV‐based screening in the Netherlands between January 1, 2017 and March 1, 2018, with cytological and histological follow‐up until August 11, 2019. The Cobas 4800 test (Roche Indianapolis, IN) was used as the HPV test, which reports whether the screening result was HPV16 and/or HPV18 positive.

### 2.3 Cancer registry data

A time window of 10 years after cancer diagnosis was used to calculate relative survival, after which we assumed no additional excess mortality. Data from the Netherlands Cancer Registry (IKNL)^19^ collected between 2005 and 2014 was used to estimate the 10‐year survival after cervical cancer detection as well as the relative survival of cancers that were missed in the first round but detected in the second round, after accounting for a possible stage shift.

### 2.4 Model‐based parameters

The probability of progression from CIN2/3 to cancer in 5 years was estimated using a previously published statistical analysis of data from the national pathology databank (PALGA).^18^ Time from CIN2/3 to cancer was described by a gamma density, with different distributions for HPV16/18‐positive and HPV16/18‐negative lesions. The transition time between cancer stage *Fédération Internationale de Gynécologie et d'Obstétrique* (FIGO) 1 and FIGO 2/3 and between FIGO 2/3 and FIGO 4 was taken from a microsimulation model that was calibrated on the age‐specific cancer stage distribution.^20^

### 2.5 Screening strategies

Table 1 summarizes the 19 triage strategies included in this analysis, which refer to HPV‐positive women either at baseline (direct referral) or after a repeat test (indirect referral). Direct referral is based on HPV genotype (either *16/18* or *7‐types*: 16/18/31/33/45/52/58), cytology or a combination of both. The seven hr types were chosen because they are covered by the nonavalent HPV vaccine^21^ and are detectable with most full genotyping assays and three commercially available extended genotyping tests.^5^, ^6^, ^7^ Indirect referral is based on repeat cytology after 6–12 months where women are referred after an abnormal (atypical squamous cells of undetermined significance [ASC‐US]/low‐grade squamous intraepithelial lesions [LSIL] or HSIL) result irrespective of HPV status. Note that in the Dutch cytological system, ASC‐H (atypical squamous cells – cannot rule out high‐grade squamous intraepithelial lesion) is classified as high‐grade cytology (HSIL).

**TABLE 1:** Overview of screening scenarios considered in the analysis.

Strategy no.	Referral by baseline genotype	Referral by baseline cytology		
		HSIL	ASC‐US/LSIL	NILM
1	Refer all	–	–	–
2	7‐types positive: Refer	7‐types negative: Refer	7‐types negative: Refer	7‐types negative: Repeat
3	7‐types positive: Refer	7‐types negative: Refer	7‐types negative: Refer	7‐types negative: Next round
4	7‐types positive: Refer	7‐types negative: Refer	7‐types negative: Repeat	7‐types negative: Repeat
5	7‐types positive: Refer	7‐types negative: Refer	7‐types negative: Repeat	7‐types negative: Next round
6	16/18 positive: Refer	16/18 negative: Refer	7‐types negative: Refer	7‐types negative: Repeat
7	16/18 positive: Refer	16/18 negative: Refer	16/18 negative: Refer	16/18 negative, 7‐types positive: Repeat 16/18 negative, 7‐types negative: Next round
8	16/18 positive: Refer	16/18 negative: Refer	16/18 negative, 7‐types positive: Refer 16/18 negative, 7‐types negative: Repeat	16/18 negative: Repeat
9	16/18 positive: Refer	16/18 negative: Refer	16/18 negative, 7‐types positive: Refer 16/18 negative, 7‐types negative: Repeat	16/18 negative, 7‐types positive: Repeat 16/18 negative, 7‐types negative: Next round
10	16/18 positive: Refer	16/18 negative: Refer	16/18 negative: Repeat	16/18 negative: Repeat
11	16/18 positive: Refer	16/18 negative: Refer	16/18 negative: Repeat	16/18 negative, 7‐types positive: Repeat 16/18 negative, 7‐types negative: Next round
12	–	Refer all	Refer all	Repeat all
13	–	Refer all	Refer all	7‐types positive: Repeat 7‐types negative: Next round
14	–	Refer all	7‐types positive: Refer 7‐types negative: Repeat	Repeat all
15	–	Refer all	7‐types positive: Refer 7‐types negative: Repeat	7‐types positive: Repeat 7‐types negative: Next round
16	–	Refer all	16/18 positive: Refer 16/18 negative: Repeat	Repeat all
17	–	Refer all	16/18 positive: Refer 16/18 negative: Repeat	7‐types positive: Repeat 7‐types negative: Next round
18	–	Refer all	Repeat all	Repeat all
19	–	Refer all	Repeat all	7‐types positive: Repeat 7‐types negative: Next round

Abbreviations: ASC‐US, atypical squamous cells of undetermined significance; HSIL, high‐grade squamous intraepithelial lesions; LSIL, low‐grade squamous intraepithelial lesions; NILM, negative for intraepithelial lesion or malignancy.

The reference strategy (Strategy 1) is the most aggressive strategy in which all HPV‐positive women are directly referred for colposcopy. Strategies 2–5 immediately refer all women positive for the 7‐types, and cytology is only performed if negative for the 7‐types. Among those 7‐types negative, those with moderate/severe cytological abnormality (HSIL) are immediately referred. Two options are considered for women who are 7‐types negative with borderline/low‐grade abnormality (ASC‐US/LSIL): (1) all referred, or (2) all repeated. Those who are 7‐types negative with normal cytology (NILM) are either all repeated or are all dismissed from follow‐up until the next screening round.

Strategies 6–11 immediately refer all HPV16/18‐positive women and manage HPV16/18‐negative women based on their cytology result. HPV16/18‐negative women with HSIL are immediately referred. Three options are considered for HPV16/18‐negative women with ASC‐US/LSIL cytology: (1) all referred, (2) referral only if 7‐types positive (besides HPV16/18) and repeat testing otherwise, or (3) repeat testing for all women. HPV16/18‐negative women with normal cytology are either all repeated or are only repeated if 7‐types positive (besides HPV16/18).

Strategies 12–19 shift to cytology‐based immediate referral, meaning no immediate referrals are made without knowledge of the cytology result. For these strategies, women with HSIL are always immediately referred. Four options are considered for managing ASC‐US/LSIL cytology: (1) all referred, (2) referral if 7‐types positive, (3) referral if HPV16/18‐positive, or (4) repeat testing. Women with normal cytology are either all repeated or are only repeated if 7‐types positive.

### 2.6 Cost and health utilities

In line with Dutch guidelines for economic evaluations,^22^ a societal perspective was adopted. Direct and indirect costs per patient associated with screening and treatment are listed in Table 2 and were taken from previously published studies and official Dutch guidelines. All costs are presented in euros (€) and were indexed to the year 2024 using the consumer price index.^23^ Costs and future effects were discounted by 3% and 1.5% per year, respectively, according to the Dutch guidelines for economic evaluations.^22^ For each strategy, the total direct costs included the costs for screening (including routine examinations, triage, and retesting) as well as costs of treatment for CIN2/3 and cervical cancer. Screening and treatment costs were taken from several studies with details given in Table S1.^9^, ^19^, ^22^, ^24^, ^25^, ^26^, ^27^, ^28^, ^29^, ^30^ Utility weights for health states (CIN2/3, cancer diagnosis, control/remission, and [pre]terminal cancer) were taken from a disease burden study.^31^ The reporting of this study complies with the Consolidated Health Economic Evaluation Reporting Standards (CHEERS) guidelines (Table S2).^32^

**TABLE 2:** Inputs for cost‐effective analysis.

Input	Value	References
Costs^a^		
First cytology test	€32	24
Repeat cytology test	€65	24
CIN0/1	€609	9, 22, 25, 26, 27, 28
CIN2 treatment + diagnosis	€1855	9, 22, 25, 26, 27, 28
CIN3 treatment + diagnosis	€2226	9, 22, 25, 26, 27, 28
Cancer	€15,095	19, 27, 29
Death	€32,555	–19, 27, 29
Health utilities (per year)^b^		
CIN2/3	0.93 (0.5 years)	31
Cancer survivor		–
Cancer diagnosis/ therapy	0.57 (0.5 years)	31
Monitor (remission/ control phase)	0.80 (3.5 years)	31
Cancer non‐survivor		–
Cancer diagnosis/ therapy	0.57 (0.5 years)	31
Monitor (remission/ control phase)	0.80 (time between diagnosis and preterminal phase^c^)	
(Pre)terminal phase	0.20 (0.5 years)	
Model‐based parameters		
Probability CIN2/3 progresses to cancer in 5 years (HPV 16/18): shape	3.33	18, 20
Probability CIN2/3 progresses to cancer in 5 years (HPV 16/18): scale	9.67	18, 20
Probability CIN2/3 progresses to cancer in 5 years (non‐HPV 16/18): shape	9.14	18, 20
Probability CIN2/3 progresses to cancer in 5 years (non‐HPV 16/18): scale	2.49	18, 20
Transition time between FIGO 1 and FIGO 2/3	8 years	17
Transition time between FIGO 2/3 and FIGO 4	4 years	17

Abbreviations: CIN (2/3+), cervical intraepithelial neoplasia (grade 2/3); FIGO, International Federation of Gynaecology and Obstetrics; HPV, human papillomavirus.

^a^ All costs indexed to 2024, more details are provided in Table S1.

^b^ Relative to a year lived in perfect health.

^c^ A 10‐year time horizon was used for cancer survival after diagnosis.

### 2.7 Statistical analysis

For each strategy, the NMB was calculated as the total number of quality‐adjusted life‐years (QALYs) gained multiplied by the Dutch threshold for preventive interventions of €20,000/QALY gained^30^ minus the total cost, and compared to the most aggressive strategy in which all women were immediately referred at baseline. The strategy with the highest NMB was considered optimal from the cost‐effectiveness viewpoint. We assumed that lesions missed in the baseline round that did not regress were detected at the next round after 5 years. The effect of missing a CIN2/3 case in the baseline round on the number of cancers in the second round depends on the time since onset of CIN2/3 at baseline, since the rate of clinical progression depends on genotype and increases with CIN2/3 duration.^18^ This information is not available from the POBASCAM study. Therefore, for each strategy, the time since onset of CIN2/3 was varied between 0 and 10 years in yearly increments and the NMB was recalculated using the probability of progression from CIN2/3 to cancer for each time since onset. Non‐parametric bootstrap of the POBASCAM data was used to estimate confidence intervals (CI) for the NMB, and the probability of a strategy having the highest NMB at each time point. All statistical analyses were performed using R software (version 4.4.3).

### 2.8 Sensitivity analysis

We conducted six one‐way sensitivity analyses regarding main analytic assumptions. (A): the time for progression from FIGO 1 to FIGO 2/3 was decreased from eight to 4 years, based on previous studies.^26^, ^33^, ^34^, ^35^, ^36^ (B): the probability of non‐regressive CIN2/3 progressing to cervical cancer was increased from the base‐case value of 0.5 to 0.7. We also checked the effect of increasing the value to 1.0. (C): a disutility for CIN0/1 of 0.01 with duration 6 months was added by taking the upper bound of the disutilities in three published studies.^24^, ^37^, ^38^ (D): the cost‐effectiveness threshold was increased from €20,000 to €50,000/QALY, as recently proposed for preventive interventions in the Netherlands.^39^ (E): the perspective was changed from a societal to a healthcare payer perspective where only direct costs were included. (F): cost‐effectiveness evaluations were conducted assuming HPV was tested on a self‐collected sample instead of a clinician‐collected sample. This increases cytology costs as positive self‐samples require an extra visit to the general practitioner (GP). The cytology costs after a positive self‐sample were set equal to those of repeat cytology.

## 3 RESULTS

### 3.1 Base‐case analysis

**FIGURE 2:**
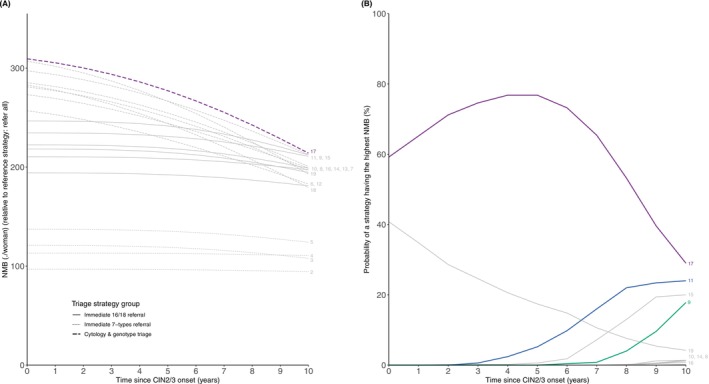
Results under the base‐case assumptions. For times since cervical intraepithelial neoplasia (grade 2/3) (CIN2/3) from 0 to 10 years, panel (A) shows the net monetary benefit of 19 human papillomavirus genotyping strategies and (B) shows the probabilistic analysis results (strategies not labeled had 0% probability of having the highest net monetary benefit (NMB) across all time points).

Figure 2A shows the NMB for each of the 19 strategies compared to the most aggressive strategy (colposcopy referral for all women at baseline) for times since CIN2/3 onset ranging between zero and 10 years. The strategy with the highest NMB was immediate referral in case of HSIL or ASC‐US/LSIL with 16/18‐positivity, and repeat cytology in case of ASC‐US/LSIL without positivity for 16/18 or normal cytology with 7‐types‐positivity (Strategy 17). The gain in NMB per woman of this strategy (compared to referring all women at baseline) decreased from €309.3 (95% CI: 245.2, 310.0) per woman at zero years since onset of CIN2/3 to €214.1 (95% CI: 115.2, 243.6) per woman at 10 years since onset of CIN2/3. This strategy led to 34,248 colposcopy referrals per 100,000 HPV‐positive women in the first round and, at 5 years since onset of CIN2/3, failed to prevent 27 cancers per 100,000 HPV‐positive women over two screening rounds (Table 3). If the time since onset of CIN2/3 is 10 years, the NMBs of Strategies 9 and 11 (which recommend direct referral of all HPV16/18‐positives) are equal to the NMB of Strategy 17.

**TABLE 3:** Total number of referrals per 100,000 human papillomavirus (HPV)‐positive women in the baseline round, net monetary benefit (NMB), number of cervical intraepithelial neoplasia (grade 2/3) (CIN2/3) and cancers detected per 100,000 HPV‐positive women over two rounds of screening if the time since CIN2/3 onset per strategy at baseline is set at 5 and 10 years.

Strategy #	Number referred (/100,000 women)	5 years since CIN2/3 onset			10 years since CIN2/3 onset		
		NMB (€/woman)	CIN2/3 detected (/100,000 women)	Cancer detected (/100,000 women)	NMB (€/woman)	CIN2/3 detected (/100,000 women)	Cancer detected (/100,000 women)
1	100,000	0.0	22,126	674	0.0	22,126	674
2	80,984	96.5	22,073	675	94.3	22,072	676
3	79,092	119.0	21,967	676	107.8	21,959	684
4	77,862	112.7	22,073	675	110.6	22,072	676
5	75,970	135.3	21,967	676	124.1	21,959	684
6	60,927	192.3	21,967	676	181.1	21,959	684
7	59,035	214.8	21,861	677	194.6	21,846	692
8	57,805	208.5	21,967	676	197.4	21,959	684
9	55,913	231.1	21,861	677	210.9	21,846	692
10	55,156	219.0	21,861	677	198.9	21,846	692
11	53,264	241.5	21,755	678	212.4	21,733	700
12	41,911	227.9	21,470	700	182.8	21,437	733
13	40,019	250.4	21,364	701	196.4	21,324	741
14	38,789	244.2	21,470	700	199.1	21,437	733
15	36,897	266.7	21,364	701	212.6	21,324	741
16	36,140	254.6	21,364	701	200.6	21,324	741
17	34,248	277.2	21,258	702	214.1	21,211	749
18	33,586	243.9	21,304	709	179.4	21,256	756
19	31,693	266.5	21,198	710	192.9	21,143	764

Probabilistic analyses (Figure 2B) showed that Strategy 17 had the highest probability of having the highest NMB for times since CIN2/3 onset up to 10 years, peaking at 75.0% at 4 years. Strategy 19, the most conservative strategy in our analysis (referring only to HSIL, repeating all ASC‐US/LSIL and only 7‐types positives normal cytology), ranked second up to 5 years since CIN2/3 onset.

### 3.2 Sensitivity analysis

**FIGURE 3:**
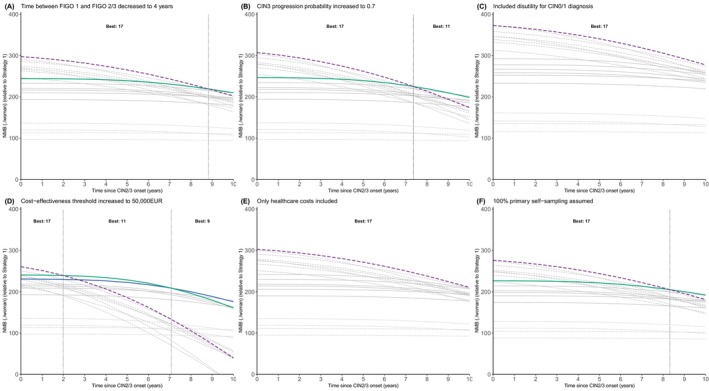
Results of one‐way sensitivity analyses for the net monetary benefit (NMB) of 19 strategies for times since onset cervical intraepithelial neoplasia (grade 2/3 or worse) (CIN2/3) from 0 to 10 years. Panels (A–F) show results of NMB for times since CIN2/3 onset ranging from 0 to 10 years when changing: (A) time between International Federation of Gynaecology and Obstetrics (FIGO) 1 and FIGO 2/3 decreased from 8 years to 4 years; (B) CIN3 progression probability increased from 0.5 to 0.7; (C) a disutility for CIN0/1 of 0.01 with duration 6 months was added; (D) cost‐effectiveness threshold increased from €20,000 to €50,000; (E) only healthcare costs included in the analysis; (F) 100% primary self‐sampling assumed (i.e., increased cost of the cytology triage test).

Sensitivity analyses showed that optimal strategies remained consistent across assumptions, though the time since onset of CIN2/3 at which one strategy overtook another in terms of highest NMB varied (Figure 3). The effect was the largest when increasing the ICER threshold or the CIN3‐to‐cancer progression probability. For these situations, Strategy 11 became optimal for time since onset of CIN2/3 between 2 and 7 years for the increased ICER threshold (Figure 3D), and after 7 years for the increased CIN3‐to‐cancer probability (Figure 3B). Strategy 11 refers all 16/18‐positive women or HSIL, with repeat testing for 16/18‐negative ASC‐US/LSIL or 7‐types positives with normal cytology. In these scenarios, for longer CIN2/3 durations, Strategy 9 had the highest NMB. This strategy also refers all 16/18‐positive women and uses a combination with cytology for other HPV genotypes.

## 4 DISCUSSION

We evaluated the cost‐effectiveness of 19 HPV genotyping strategies for the management of HPV‐positive women in cervical screening programs. These strategies referred women for colposcopy based either on HPV genotype, cytology, or a combination of both. All included strategies immediately referred women with HSIL cytology if they were not already referred based on genotype. Strategies were studied as a function of time since CIN2/3 onset at baseline ranging from 0 to 10 years, as the duration of prevalent CIN2/3 is unknown at the time of triage testing.

At a €20,000/QALY cost‐effectiveness threshold, our results showed that referral based on HPV16/18 combined with cytology improves the cost‐effectiveness by identifying hr women and that 7‐types genotyping improves the cost‐effectiveness by identifying low‐risk women that can return to screening without follow‐up (Strategy 17). If the time since onset of CIN2/3 approaches 10 years, referring all HPV16/18 positive women (Strategies 9 and 11) is also cost‐effective as the NMBs of those strategies become equal to that of Strategy 17. If a more liberal cost‐effectiveness threshold of €50,000/QALY is used as recently recommended, Strategies 9 and 11 are already cost‐effective when the time since CIN2/3 onset is 2 years.

Our findings can be applied to an HPV‐based screening program with 5‐year intervals as CIN2/3 typically develops as the result of an HPV infection. Therefore, Strategy 17 is recommended for women with a negative HPV test in the previous round as this strategy is optimal for CIN2/3 for all times since onset less than 10 years. Strategy 17 is similar to the Dutch program adopted in mid‐2022, where only HPV16/18‐positive women with ASC‐US/LSIL cytology are referred for colposcopy, with others retested after 12 months. The only difference is that Strategy 17 uses 7‐types genotyping to identify women with normal cytology eligible for repeat testing, while the rest return to routine screening. While this may add some complexity to the screening program both operationally and in terms of communication with women, no issues were observed when the Netherlands added 16/18 genotyping for women with ASC‐US/LSIL cytology. Furthermore, Sweden recently changed their triage protocol and dismisses HPV‐positive women over 32 years old with normal cytology who are negative for the 7‐types from short‐term repeat testing. This recommendation is supported by a Swedish study that found HPV types 16/18/31/33/45/52 in 85.3% of invasive cervical cancers between 2002 and 2011, with other hr types only increasing prevalence by 1.5%.^40^ To our knowledge, no other country dismisses HPV‐positive cytologically normal women from further follow‐up based on their genotype.

Additionally, immediate referral of 16/18 positive results (Strategies 9 and 11) after a new HPV positive result seems warranted if the cost‐effectiveness threshold is increased to €50,000/QALY because it is likely that most prevalent CIN2/3 cases have persisted for at least 2 years. Several countries have already implemented immediate referral based on HPV16/18 without cytology.^15^, ^41^ The United States recommends immediate colposcopy for HPV16/18‐positives, using reflex cytology to expedite treatment by offering immediate treatment to patients with 16/18‐positive HSIL cytology without a preceding confirmatory biopsy.^14^ In Australia, all HPV16/18‐positives (regardless of cytology) are also referred for colposcopy to determine if a biopsy (and treatment) is required.^42^

As vaccinated cohorts enter screening programs, HPV16/18 and HPV16/18/31/33/45/52/58 positivity rates are expected to decline substantially. This decline extends to unvaccinated individuals due to indirect protection of the bivalent HPV vaccine for types 16/18/31/45,^43^ especially with gender‐neutral vaccination. As overall HPV risk decreases, less intensive screening and triage strategies are needed to maintain cost‐effectiveness in vaccinated cohorts.^44^ Triage strategies that stratify by HPV vaccine genotypes are expected to be less sensitive to changes in the disease prevalence than cytological triage, since it is an objective test and maintaining high‐quality cytology will be challenging when the disease becomes rare. Furthermore, the demand for cytology will decrease as the HPV prevalence decreases, and it will become difficult to maintain a workforce of cytotechnicians, leading to logistical challenges. Another reason why genotyping will provide a more resilient triage program is that the risk of CIN3+ in genotype subgroups will not be affected by vaccination when vaccination only changes the distribution of genotype subgroups. Therefore, by first stratifying according to vaccine genotype subgroups, triage strategies can be developed with stratum‐specific CIN3+ risks that are robust against the effect of vaccination. Nonetheless, continued surveillance in vaccinated cohorts is still important to ensure a cost‐effective screening program.

Further, several countries now offer self‐sampling for cervical screening. In mid‐2023, the Netherlands implemented primary self‐sampling for all women invited to screening. Our results were consistent even when assuming 100% uptake of primary self‐sampling, supporting cytology as a triage test after HPV self‐sampling. In future screening, host cell DNA methylation^45^ may be considered instead of cytology to complement HPV genotyping, since they can also be evaluated directly on self‐samples.

This study has several strengths. First, our analysis is a cost‐effectiveness analysis using NMB as a metric, which is more comprehensive than the CIN3+ risk used in previous studies and allows a comparison with other health interventions incorporating both health gains and harms. Further, unlike other studies, we did not evaluate the cost‐effectiveness by means of a microsimulation model for HPV carcinogenesis. Instead, we employed a data‐driven modeling approach based on a large HPV screening cohort and screening and cancer registries, combined with a statistical progression model to bridge CIN2/3 to cancer.^18^ This allowed us to assess a trial‐based cost‐effectiveness using the predictive values of triage strategies rather than having to make assumptions about latent transitions between precancerous states, which may be oversimplified in microsimulation models and are difficult to estimate. The POBASCAM study was ideally suited for this purpose, consisting of 44,000 women with full genotyping for all HPV‐positive samples. HPV‐positive women with normal and ASC‐US/LSIL cytology were conservatively managed with repeat testing, providing a unique opportunity to evaluate conservative genotyping triage strategies.

This study also has several limitations. First, in the POBASCAM study, not all women had repeat test results. For CIN3+ cases with abnormal cytology that violated the protocol (i.e., CIN3+ detected before or without a repeat test), we imputed repeat cytology using the distribution of available repeat results in CIN3+ cases with abnormal cytology detected in the first round. Second, indirect costs for cervical cancer and death cases were not available in the Netherlands, so costs were estimated from a Germany study adjusted for purchasing power parity. Direct costs for cancer and death cases were comparable between the countries, so this is unlikely to substantially affect results. Third, our findings apply to the Dutch setting where screening is between ages 30 and 65, so generalizability may be limited to other age groups, particularly those under age 30. We also did not evaluate age‐based triage strategies that consider age, HPV genotype and cytology combined. Fourth, we assumed that lesions missed in the baseline round were detected in the next screening round. This means that our method can be assumed as a two screening round approximation of the difference in NMB between strategies. Health and cost differences between strategies may become larger when a conservative strategy is maintained for multiple screening rounds. However, triage strategies for the second screening round are currently being evaluated. It is likely that these will depend on HPV results from the first round because HPV type persistence over two screening rounds is strongly associated with CIN3+ risk.^46^ Finally, data from the POBASCAM trial is over 20 years old, but despite the time difference, the distribution of baseline cytology results and HPV genotype in HPV‐positive women are fairly similar between the POBASCAM study and the 2017 primary HPV national screening program. For example, the percentages of ASC‐US/LSIL and HSIL were 17% and 14% in the POBASCAM study compared to 21% and 11% in the national program, while HPV16/18 percentages were 43% in POBASCAM and 34% in the national program.

## 5 CONCLUSION

HPV genotyping in combination with cytology is cost‐effective for triage of HPV‐positive women. As cervical screening continues to evolve with new technologies, more tailored triage strategies such as these are essential for maintaining the effectiveness and efficiency of screening programs. However, continued evaluation will be necessary as the increasing participation of HPV‐vaccinated women shifts genotype and disease prevalence.

## AUTHOR CONTRIBUTIONS

**Kelsi R. Kroon:** Conceptualization; methodology; formal analysis; visualization; writing – original draft. **Johannes A. Bogaards:** Conceptualization; supervision; writing – review and editing. **Johannes Berkhof:** Conceptualization; supervision; writing – review and editing.

## FUNDING INFORMATION

This work was supported by the Horizon 2020 research and innovation program of the European Commission (RISCC project, grant agreement no. 847845) (to Kelsi R. Kroon, Johannes A. Bogaards, and Johannes Berkhof). The funders had no role in study design, data collection and analysis, decision to publish, or preparation of the manuscript.

## CONFLICT OF INTEREST STATEMENT

Kelsi R. Kroon, Johannes A. Bogaards, and Johannes Berkhof declare no conflicts of interest.

## ETHICS STATEMENT

The POBASCAM trial (Trial registration ID: NTR218) was approved by the Medical Ethics Committee of the VU University Medical Centre (Amsterdam, The Netherlands; no. 96/103) and the Ministry of Public Health (The Hague, The Netherlands; VWS no. 328650). All participants provided written informed consent.

## Supporting information


**Data S1.** Supporting Information.

## Data Availability

R code is publicly available on GitHub (https://github.com/kelsikroon/GenotypingCEA). Other data that support the findings of this study are available from the corresponding author upon request.
