# Supplementary material for: Cost‐effectiveness analysis of human papillomavirus (HPV) genotyping strategies for management of HPV‐positive women in cervical cancer screening
Source: Int J Cancer. 2026 Jan 28;158(12):3141–51. doi: 10.1002/ijc.70344 (PMC13106908; doi:10.1002/ijc.70344)
Supplement: Supplementary file 1 — Data S1. Supporting Information. [file IJC-158-3141-s001.pdf]

Supplementary Material

Cost-effectiveness analysis of human papillomavirus (HPV) genotyping strategies for management of HPV-positive women in cervical cancer screening

Kelsi R Kroon, Johannes A Bogaards, Johannes Berkhof

Table of Contents

Supplementary Table S1. *Detailed breakdown of direct and indirect costs for CIN0/1 diagnosis, CIN2/3 diagnosis and treatment, cancer and death* **... 2**

Supplementary Table S2. *The CHEERS Checklist* **.. 3**

Supplementary Table S1. Detailed breakdown of direct and indirect costs for CIN0/1 diagnosis, CIN2/3 diagnosis and treatment, cancer and death

Cost category		# per patient	Cost per medical procedure (€)	Cost per patient (€)	References
**CIN0**					
Direct costs	Colposcopies	1.37	191.7 (first) , 158.8 (repeat)	251	[9,25]
	Biopsies	1.37	70.1	96	[9,25]
	Treatment (LLETZ)	0	701	0	[9,24,25,27,28]
	Cytology	0.67	At GP office (104)	70	Average of 3 lab prices
Indirect costs	Travel to outpatient clinic	1.37	5.8 (0.26/km for 7.1km + 3.92 parking)	8	[28]
**Total costs (indexed to 2024)**				**439**	
**CIN1**					
Direct costs	Colposcopies	1.37	191.7 (first), 158.8 (repeat)	251	[9,25]
	Biopsies	1.37	70.1	96	[9,25]
	Treatment (LLETZ)	0	701	0	[9,24,25,27,28]
	Co-tests (cytology + HPV) after treatment	1.805	339 (outpatient clinic), 250 (GP office)	523	Average of 3 lab prices
Indirect costs	Travel to outpatient clinic	2.17	5.8 (0.26/km for 7.1km + 3.92 parking)	13	[28]
**Total costs (indexed to 2024)**				**913**	
**Combined CIN0/1 according to observed proportion**				**609**	
**CIN2**					
Direct costs	Colposcopies	2.01	191.7 (first), 158.8 (repeat)	352	[9,25]
	Biopsies	2.01	70.1	141	[9,25]
	Treatment (LLETZ)	0.75	701	526	[9,24,25,27,28]
	Co-tests (cytology + HPV) after treatment	2.55	339 (outpatient clinic), 250 (GP office)	776	Average of 3 lab prices
Indirect costs	Travel to outpatient clinic	4.31	5.8 (0.26/km for 7.1km + 3.92 parking)	25	[28]
**Total costs (indexed to 2024)**				**1855**	
**CIN3**					
Direct costs	Colposcopies	2.30	191.7 (first), 158.8 (repeat)	398	[9,25]
	Biopsies	2.30	70.1	161	[9,25]
	Treatment (LLETZ)	1.15	701	806	[9,24,25,27,28]
	Co-tests (cytology + HPV) after treatment	2.59	339 (outpatient clinic), 250 (GP office)	789	Average of 3 lab prices
Indirect costs	Travel to outpatient clinic	5.04	5.8 (0.26/km for 7.1km + 3.92 parking)	29	[28]
**Total costs (indexed to 2024)**				**2226**	
**Cancer**					
Direct costs (2010)				8000	[26]
Indirect costs (2011)				2792	[18,30]
**Total costs (indexed to 2024)**				**15095**	
**Death**					
Direct costs (2010)				19600	[26]
Indirect costs (2011)				3729	[18,30]
**Total costs (indexed to 2024)**				**32555**	

Supplementary Table S2. The CHEERS Checklist

Topic	No.	Item	Location where item is reported
**Title**			
Title	1	Identify the study as an economic evaluation and specify the interventions being compared.	Title, page 1
**Abstract**			
Abstract	2	Provide a structured summary that highlights context, key methods, results, and alternative analyses.	Abstract, page 2
**Introduction**			
Background and objectives	3	Give the context for the study, the study question, and its practical relevance for decision making in policy or practice.	Introduction, paragraphs 2 and 3
**Methods**			
Health economic analysis plan	4	Indicate whether a health economic analysis plan was developed and where available.	Not reported
Study population	5	Describe characteristics of the study population (such as age range, demographics, socioeconomic, or clinical characteristics).	Materials and methods, subsection "Screening cohort data"
Setting and location	6	Provide relevant contextual information that may influence findings.	Materials and methods, first paragraph
Comparators	7	Describe the interventions or strategies being compared and why chosen.	Materials and methods, subsection "Screening strategies"
Perspective	8	State the perspective(s) adopted by the study and why chosen.	Materials and methods, subsection "Cost and health utilities"
Time horizon	9	State the time horizon for the study and why appropriate.	Materials and methods, subsection "Cancer registry data"
Discount rate	10	Report the discount rate(s) and reason chosen.	Materials and methods, subsection "Cost and health utilities"
Selection of outcomes	11	Describe what outcomes were used as the measure(s) of benefit(s) and harm(s).	Materials and methods, subsection "Cost and health utilities"
Measurement of outcomes	12	Describe how outcomes used to capture benefit(s) and harm(s) were measured.	Materials and methods, subsection "Statistical analysis"
Valuation of outcomes	13	Describe the population and methods used to measure and value outcomes.	Materials and methods, subsection "Cost and health utilities" and Table 2 (Health Utilities)
Measurement and valuation of resources and costs	14	Describe how costs were valued.	Materials and methods, subsection "Cost and health utilities" and Table 2 (Costs)
Currency, price date, and conversion	15	Report the dates of the estimated resource quantities and unit costs, plus the currency and year of conversion.	Materials and methods, subsection "Cost and health utilities" and Table 2, Table S2
Rationale and description of model	16	If modelling is used, describe in detail and why used. Report if the model is publicly available and where it can be accessed.	Materials and methods, subsection "Model-based parameters" and "Statistical analysis"

Analytics and assumptions	17	Describe any methods for analysing or statistically transforming data, any extrapolation methods, and approaches for validating any model used.	Materials and methods, subsection “Statistical analysis” and Figure S1
Characterising heterogeneity	18	Describe any methods used for estimating how the results of the study vary for subgroups.	Not reported
Characterising distributional effects	19	Describe how impacts are distributed across different individuals or adjustments made to reflect priority populations.	Not reported
Characterising uncertainty	20	Describe methods to characterise any sources of uncertainty in the analysis.	Materials and methods, subsection “Statistical analysis”
Approach to engagement with patients and others affected by the study	21	Describe any approaches to engage patients or service recipients, the general public, communities, or stakeholders (such as clinicians or payers) in the design of the study.	Not reported
**Results**			
Study parameters	22	Report all analytic inputs (such as values, ranges, references) including uncertainty or distributional assumptions.	Materials and methods, subsection “Model-based parameters” and Table 2
Summary of main results	23	Report the mean values for the main categories of costs and outcomes of interest and summarise them in the most appropriate overall measure.	Results, subsection “Base-case analysis”
Effect of uncertainty	24	Describe how uncertainty about analytic judgments, inputs, or projections affect findings. Report the effect of choice of discount rate and time horizon, if applicable.	Results, subsection “Sensitivity analysis”
Effect of engagement with patients and others affected by the study	25	Report on any difference patient/service recipient, general public, community, or stakeholder involvement made to the approach or findings of the study	Not reported
**Discussion**			
Study findings, limitations, generalisability, and current knowledge	26	Report key findings, limitations, ethical or equity considerations not captured, and how these could affect patients, policy, or practice.	Discussion
**Other relevant information**			
Source of funding	27	Describe how the study was funded and any role of the funder in the identification, design, conduct, and reporting of the analysis	End of manuscript, funding statement
Conflicts of interest	28	Report authors conflicts of interest according to journal or International Committee of Medical Journal Editors requirements.	End of manuscript, Conflict of Interest statement
